# Nomograms to Predict the Density of Tumor-Infiltrating Lymphocytes in Patients With High-Grade Serous Ovarian Cancer

**DOI:** 10.3389/fonc.2021.590414

**Published:** 2021-02-25

**Authors:** Danian Dai, Lili Liu, He Huang, Shangqiu Chen, Bo Chen, Junya Cao, Xiaolin Luo, Feng Wang, Rongzhen Luo, Jihong Liu

**Affiliations:** ^1^ Department of Gynecology and Obstetrics, The Fifth Affiliated Hospital of Sun Yat-Sen University, Zhuhai, China; ^2^ Department of Gynecologic Oncology, State Key Laboratory of Oncology in South China, Collaborative Innovation Center for Cancer Medicine, Sun Yat-sen University Cancer Center, Guangzhou, China; ^3^ Department of Pathology, State Key Laboratory of Oncology in South China, Collaborative Innovation Center for Cancer Medicine, Sun Yat-sen University Cancer Center, Guangzhou, China; ^4^ Department of Breast Cancer, Cancer Center, Guangdong Provincial People's Hospital and Guangdong Academy of Medical Sciences, Guangzhou, China

**Keywords:** high-grade serous ovarian cancer, nomograms, tumor-infiltrating lymphocytes, tumor microenvironment, blood indicators

## Abstract

**Background:**

Tumor-infiltrating lymphocytes (TILs) have important roles in predicting tumor therapeutic responses and progression, however, the method of evaluating TILs is complicated. We attempted to explore the association of TILs with clinicopathological characteristics and blood indicators, and to develop nomograms to predict the density of TILs in patients with high-grade serous ovarian cancer (HGSOC).

**Methods:**

The clinical profiles of 197 consecutive postoperative HGSOC patients were retrospectively analyzed. Tumor tissues and matched normal fallopian tubes were immunostained for CD3+, CD8+, and CD4+ T cells on corresponding tissue microarrays and the numbers of TILs were counted using the NIH ImageJ software. The patients were classified into low- or high-density groups for each marker (CD3, CD4, CD8). The associations of the investigated TILs to clinicopathological characteristics and blood indicators were assessed and the related predictors for densities of TILs were used to develop nomograms; which were then further evaluated using the C-index, receiver operating characteristic (ROC) curves and calibration plots.

**Results:**

Menopausal status, estrogen receptor (ER), Ki-67 index, white blood cell (WBC), platelets (PLT), lactate dehydrogenase (LDH), and carbohydrate antigen 153 (CA153) had significant association with densities of tumor-infiltrating CD3+, CD8+, or CD4+ T cells. The calibration curves of the CD3+ (C-index = 0.748), CD8+ (C-index = 0.683) and CD4+ TILs nomogram (C-index = 0.759) demonstrated excellent agreement between predictions and actual observations. ROC curves of internal validation indicated good discrimination for the CD8+ TILs nomogram [area under the curve (AUC) = 0.659, 95% CI 0.582–0.736] and encouraging performance for the CD3+ (AUC= 0.708, 95% CI 0.636–0.781) and CD4+ TILs nomogram (AUC = 0.730, 95% CI 0.659–0.801).

**Conclusion:**

Menopausal status, ER, Ki-67 index, WBC, PLT, LDH, and CA153 could reflect the densities of T cells in the tumor microenvironment. Novel nomograms are conducive to monitor the immune status of patients with HGSOC and help doctors to formulate the appropriate treatment strategies.

## Introduction

Ovarian cancer is the most lethal gynecological malignancy worldwide (1); of which high-grade serous ovarian cancer (HGSOC) accounts for 70–80% of all ovarian cancer-related deaths. The prognosis of HGSOC has not been significantly improved in the past decades (2). Clinically, HGSOC prognosis is mainly assessed using the International Federation of Gynecology and Obstetrics (FIGO) staging system (3). However, the predictive limitations of the FIGO staging system have forced researchers to explore more specific and accurate prediction models using histological classification, molecular typing, biomarkers, and tumor-infiltrating lymphocytes (TILs) approaches (4–8).

TILs refer to mononuclear immune cells (such as white blood cells [WBC], T-cells and B-cells) nested in the tumor stroma or intra-epithelium (9). It has now been well-established that TILs play a crucial role in controlling tumor growth, recognition of cancer antigens, therapeutic response, and the inhibition of cancer development in solid tumors (10, 11). The survival benefits of TILs have been shown in a variety of cancers including, but not limited to, melanoma (12), colon cancer (13), and ovarian cancer (14). Quantification of TILs has shown promising potential to be used as a new biomarker for cancer. Approaches of quantifying TILs include image-analysis, methylation signature and multi-omics data, and have been devised to assess therapeutic prediction or prognosis (15–17). However, there is not a convenient method to provide standardized and efficient TILs evaluation in clinical practice yet.

Blood routine and biochemical indicators are widely used to monitor the patients’ health conditions because of related cancer-induced disorders in the bio-energetic metabolism, and their potential roles in tumors have been extensively studied in recent years. Blood indicators, including lactate dehydrogenase (LDH) (18), the NLR (neutrophil-to-lymphocyte ratio) (19), the PLR (platelet-to-lymphocyte ratio) (20), the LMR (lymphocyte-to-monocyte ratio) (21), carbohydrate antigen 125 (CA125) (22), and carbohydrate antigen 153 (CA153) (23), have shown important diagnostic and/or prognostic value in ovarian cancer. Additionally, nomograms, considered as a clinically easy-to-implement and reliable calculating model, have been established by combining related risk factors to help clinicians to develop individualized treatment and follow-up management strategies in breast, gastric and bladder cancers (24–27). These have attracted our attention to devise such a model for HGSOC.

To the best of our knowledge, no nomograms had been proposed for predicting TILs in HGSOC. This study aimed to explore the association of TILs (CD3+, CD8+, and CD4+) to the clinicopathological characteristics and blood indicators of HGSOC; based on which nomograms were established to assess the density levels of TILs, with the hope of providing a convenient method to monitor the immune status of HGSOC patients and help to guide therapeutic strategies in clinical practice.

## Materials and Methods

### Patients

One hundred ninety-seven surgically resected ovarian cancer samples and matched normal fallopian tubes were collected at the Sun Yat-sen University Cancer Center between February 1, 2008, and December 31, 2013. All the included patients were histologically diagnosed as HGSOC. Additionally, no patients had a second primary tumor, chronic inflammatory disease (such as autoimmune disease and infection, etc.), or received any preoperative treatments, including chemotherapy, radiotherapy, targeted therapy, immunotherapy. Basic clinicopathological data of the patients were obtained by reviewing their medical records. This study was approved by the Institutional Review Board and Ethics Committee at Sun Yat-sen University Cancer Center.

### Laboratory Measurements of Blood Biochemical Indicators

Tumor biomarkers, including CA125, CA153, carbohydrate antigen 199 (CA199), and carcinoembryonic antigen (CEA), were measured using an automatic electrochemistry luminescence immunoassay system [ROCHE E170 (Roche, Mannheim, Germany)]. WBC, neutrophils, lymphocytes, monocytes, platelets (PLT), NLR, PLR, LMR, LDH, albumin (ALB), and C-reactive protein (CRP) were classified as inflammatory markers. WBC, neutrophils, lymphocytes, monocytes, and PLT were measured by routine blood examination [XE-5000TM Automated Hematology System (Sysmex UK Ltd., Milton Keynes, UK)]. LDH, ALB, and CRP were tested with a blood analyzer [Hitachi Automatic Analyzer 7600-020 (Hitachi, Tokyo, Japan)]. All biomarkers data were obtained within one week before surgery. The normal ranges of CA125, CA153, CA199, CEA, WBC, neutrophils, lymphocytes, monocytes, PLT, LDH, ALB, and CRP levels in blood were 0–35 U/mL, 0–25 U/L, 0–35 U/mL, 0–5 ng/mL, 3.69–9.16 10E9/L, 2.0–7.0 10E9/L, 0.8–4 10E9/L, 0.12–1.2 10E9/L, 100–300 10E9/L, 109–245 U/L, 35–55 g/L, and 0–8.2 mg/L, respectively. NLR, PLR, and LMR had no standard normal range. The patients were classified into four subgroups according to quartiles of CA153 (quartile1, 22.3; quartile2, 62.1; quartile3, 148.1), WBC (quartile1, 5.95; quartile2, 7.2; quartile3, 8.8), PLT (quartile1, 234.7; quartile2, 305.0; quartile3, 379.8) and LDH (quartile1, 182.1; quartile2, 224.5; quartile3, 306.6).

### Tissue Microarray (TMA) Construction and Immunohistochemistry (IHC)

The tissue array (TMA) slides contained 197 pairs of HGSOC cases and matched normal fallopian tubes. Each core tissue biopsy (1 mm in diameter) was taken from individual paraffin-embedded HGSOC or internal controls (donor block) and re-arranged in a new recipient paraffin block (tissue array block) with a tissue array instrument (Minicore Excilone, Minicore, UK). Then, the paraffin-embedded tissue specimens were cut into 4-mm sections and mounted onto glass slides. The slides were stained with anti-CD3 (2GV6; 1:100; Roche/Ventana), -CD8 (AM0063; 1:100; Ascend Biotechnology Co.,Ltd), -CD4 (ZM-0418; 1:100; ZSGB-BIO), -Ki-67 (ZA-0502; 1:100; ZSGB-BIO), -p53 (Bp-53-12; 1:100; BioGenex), -ER (clone SP1; Roche/Ventana), and all slides were stained with hematoxylin and eosin (H&E). Stained slides from representative areas of the core of the tissue biopsy were scanned using an Olympus digital slide scanner. Each slide was evaluated by two pathologists, who were blinded to the clinical status of the patients. Densities of CD3+, CD8+, and CD4+ TILs per mm^2^ were calculated using the NIH ImageJ v1.48 software, a Java-based image processing program (28). Patients were divided into subgroups based on each immunostained marker. The median density of TILs was chosen as the cut-off value for defining high and low expression. ER positivity threshold was defined as ≥1% displaying nucleus ER staining of any intensity (29). A median Ki-67 index of 30% was chosen as the cut-off value for defining high and low Ki-67 index. For p53, tumors with more than 60% immunoreactivity in the nuclei were defined as mutational, otherwise wild.

### Construction and Validation of Nomograms

Independent predictors of CD3+, CD8+, and CD4+ TILs for HGSOC patients were identified by univariate and multivariate analyses. All variables were evaluated with the backward multivariate binary logistic regression model (30). Then, based on the screened variables, three nomograms were developed. Bootstrapping with 40 resamples were applied for internal validation of the nomograms. The performance of each nomogram for prediction was judged using the Harrell's concordance index (C-index) and receiver operating characteristic (ROC) curves. Calibration curves were implemented to validate the accuracy and reliability of the nomograms (31).

### Statistical Analysis

Statistical analyses were performed using the SPSS software, version 22.0 (SPSS, Chicago, IL, USA) and the programming language R (version 3.6.3, http://www.R-project.org) for Windows. The correlation between the clinical variables and density levels of TILs (CD3+, CD8+, CD4+ T cells) were assessed by the chi-squared test. Based on the levels of TILs, all blood indicators between subgroups were displayed as median (minimum–maximum) and the distribution differences were analyzed by non-parametric tests. Due to non-normal distributions of blood indicators, the association between blood indicators and the expression levels of TILs were assessed using the Spearman's correlation test to obtain correlation coefficients. All variables with p less than 0.1 in the univariate analysis were incorporated into multivariate analyses to identify the independent predictors related to TILs. According to the results of the multivariate analysis, nomograms, ROC curves, and calibration plots were established respectively by R 3.6.3 with the *rms, ROC*, and *calibrate* packages. All statistical tests were two-sided, and *p* values less than 0.05 were considered statistically significant.

## Results

### Patients' Clinical Characteristics 

A total of 197 HGSOC patients were found eligible for this study and their characteristics are detailed in **Table 1**. The patients’ age ranged from 22 to 85 years, with a median age of 52 years. 111 (56.3%) patients were in menopause. Most of the tumors recorded were larger than 5 cm [n = 165 (83.8%)] and occurred in the bilateral ovaries [n = 144 (73.1%)]. The pathological differential for the vast majority of tumors was poorly differentiated (160, 81.2%), and only 37 patients were moderately differentiated. 143 (72.6%) patients were classified as stage III–IV (2009 FIGO) and 144 (73.1%) patients had ascites. Approximately 1/3 of the patients had metastatic lymph nodes and 57 (28.9%) patients did not undergo lymphadenectomy. 173 (87.8%) cases had p53 mutations, and 6 cases without successful immunohistochemical (IHC) staining. Hormone levels were also tested as follows: 161 (81.7%) for positive ER and 128 (65.0%) for positive progesterone receptor (PR). Using a Ki-67 index of 30% as boundary, the amount of patients in the two groups is similar.

**Table 1:** Basic clinicopathological characteristics of 197 high-grade serous ovarian cancer patients.

Variables	No. of patients (%)
Age (year)	
<52	96 (48.7%)
≥52	101 (51.3%)
Menopausal status	
Negative	86 (43.7%)
Positive	111 (56.3%)
Tumor size (cm)	
<5	32 (16.2%)
≥5	165 (83.8%)
Location	
Unilateral	53 (26.9%)
Bilateral	144 (73.1%)
p53 status	
Wild	18 (9.1%)
Mutant	173 (87.8%)
Unknown	6 (3.1%)
Pathological differentiation	
Moderate	37 (18.8%)
Poor	160 (81.2%)
ER	
Negative	36 (18.3%)
Positive	161 (81.7%)
PR	
Negative	69 (35.0%)
Positive	128 (65.0%)
Ki-67 index (%)	
<30	93 (47.2%)
≥30	104 (52.8%)
Ascites	
No	53 (26.9%)
Yes	144 (73.1%)
Lymph node metastasis	
Negative	78 (39.6%)
Positive	62 (31.5%)
Unknown	57 (28.9%)
FIGO stage (2009)	
I	19 (9.6%)
II	35 (17.8%)
III	118 (59.9%)
IV	25 (12.7%)

ER, estrogen receptor; PR, Progesterone receptor.

### Immunohistochemical Characteristics of Various Markers

**Figure 1:**
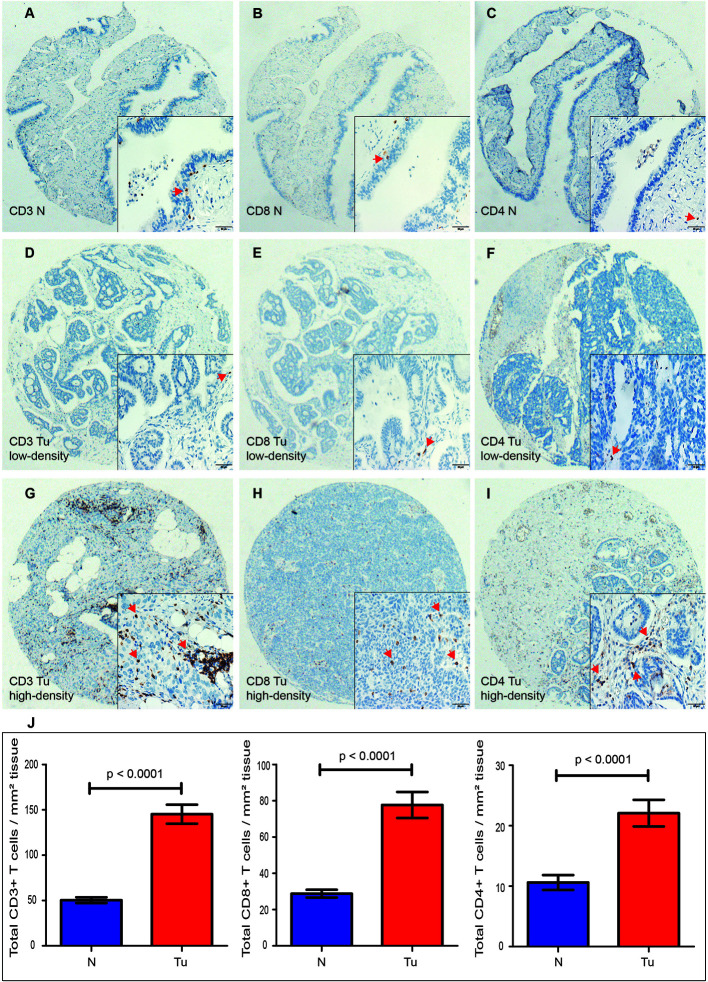
Expression of CD3+, CD8+, and CD4+ TILs in HGSOC tissues (Tu) and matched normal epithelium of the fallopian tubes (N), shown at 40× magnification with inset (400×). **(A–C)** Representative images of CD3+, CD8+, and CD4+ TILs in the normal fallopian tube tissues. **(D, G)** Representative images of low-density and high-density of CD3+ TILs in the HGSOC tissues. **(E, H)** Representative images of low-density and high-density CD8+ TILs in the HGSOC tissues. **(F, I)** Representative images of low-density and high-density of CD4+ TILs in the HGSOC tissues. **(J)** Box plots of CD3+, CD8+, and CD4+ TILs per mm^2^ in tumor tissues (n=197) or normal epithelium of the fallopian tubes (n=173). Quantitative data are presented as mean ± SEM. TILs, tumor-infiltrating lymphocytes; HGSOC, high-grade serous ovarian cancer.

**Figure 2:**
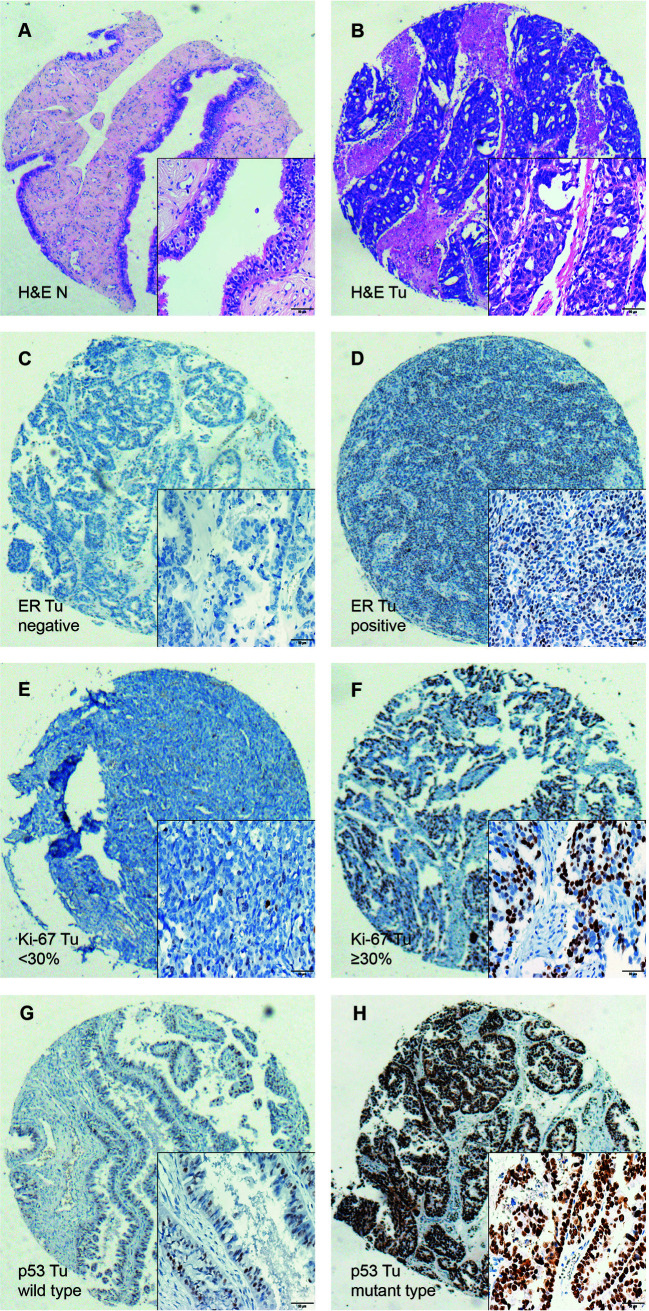
Immunohistochemical staining of H&E, ER, Ki-67, and p53 in HGSOC tissues (Tu) and matched normal fallopian tubes tissue (N), shown at 40× magnification with inset (400×). **(A, B)** Representative images of H&E in the matched normal epithelium of the fallopian tubes (N) and HGSOC tissues (Tu). **(C, D)** Representative images of negative and positive ER in HGSOC tissues are presented. **(E, F)** Representative images of high and low Ki-67 index in HGSOC tissues are shown **(G, H)** Representative images of mutant type and wild type p53 staining in HGSOC tissues are presented. TILs, tumor-infiltrating lymphocytes; HGSOC, high-grade serous ovarian cancer; H&E, Hematoxylin and Eosin.

TILs were examined in 197 pairs of tissue samples from patients with HGSOC and normal fallopian tube. As 24 out of the 197 matched normal specimens lack the fallopian tube epithelium, the actual number of normal specimens was 173. We detected the density levels of CD3+, CD8+, and CD4+ T cells /mm^2^ in tumor and normal specimens, respectively (**Figures 1A–C**). The degree of various T cell infiltrations in the tumors was significantly higher than that of the normal tissues (all p < 0.001) (**Figure 1J**). The median density of CD3+ T cells in the tumor was 104/mm^2^ (1/mm^2^–791/mm^2^), 48/mm^2^ (0/mm^2^–684/mm^2^) for CD8+ T cells, and 12/mm^2^ (0/mm^2^–236/mm^2^) for CD4+ T cells, respectively. Low-density level was defined as a value below the median, and high-density level was defined as a value above the median (**Figures 1D–I**). Representative H&E and IHC images of ER, Ki-67 index, and p53 are shown in **Figures 2A–H**, respectively.

### Association Between Clinical Characteristics and Tumor-Infiltrating T Cells

The association between CD3+, CD4+, CD8+ TILs, and clinicopathological characteristics is shown in **Table 2**. CD3+, CD4+, CD8+ TILs were significantly correlated with tumor differentiation and ER status (all p < 0.05). Additionally, CD8+ TILs were relatively higher in older patients with more than 52 years (57.5% vs. 42.6%, p = 0.039) and menopausal patients (58.6% vs. 41.1%, p = 0.008). Only the level of CD4+ TILs were higher in patients with high Ki-67 index (62.5% vs. 37.5%, p < 0.001). Tumor size, location, ascites, and lymph node metastasis had no significant association with the densities of CD3+, CD8+, and CD4+ TILs.

**Table 2:** Association between clinicopathological characteristics and tumor infiltrating T cells.

Variable	N=197	CD3+ T cells			CD8+ T cells			CD4+ T cells		
		Low	High	p-value^a^	Low	High	p-value^a^	Low	High	p-value^a^
Age(year)				0.175			0.039*			0.522
<52 (young)	96	53 (55.2%)	43 (44.8%)		55 (57.3%)	41 (42.7%)		50 (52.1%)	46 (47.9%)	
≥52 (older)	101	46 (45.5%)	55 (54.5%)		43 (42.6%)	58 (57.4%)		48 (47.5%)	53 (52.5%)	
Menopausal satus				0.097			0.008*			0.074
Negative	86	49 (57.0%)	37 (43.0%)		52 (60.5%)	34 (39.5%)		49 (57.0%)	37 (43.0%)	
Positive	111	50 (45.0%)	61 (55.0%)		46 (41.4%)	65 (58.6%)		49 (44.1%)	62 (55.9%)	
Tumor size(cm)				0.676			0.975			0.459
<5	32	15 (46.9%)	17 (53.1%)		16 (50.0%)	16 (50.0%)		14 (43.8%)	18 (56.3%)	
≥5	165	84 (50.9%)	81 (49.1%)		82 (49.7%)	83 (50.3%)		84 (50.9%)	81 (49.1%)	
Location				0.838			0.838			0.907
Unilateral	53	26 (49.1%)	27 (50.9%)		27 (50.9%)	26 (49.1%)		26 (49.1%)	27 (50.9%)	
Bilateral	144	73 (50.7%)	71 (49.3%)		71 (49.3%)	73 (50.7%)		72 (50.0%)	72 (50.0%)	
p53 status				0.671			0.357			0.705
Wild	18	8(44.4%)	10 (55.6%)		7 (38.9%)	11 (61.1%)		8(44.4%)	10(55.6%)	
Mutant	173	86 (49.7%)	87 (50.3%)		87 (50.3%)	86 (49.7%)		85 (49.1%)	88 (50.9%)	
Unknown	6									
Pathological differentiation				0.049*			0.041*			0.016*
Moderate	37	24 (64.9%)	13 (35.1%)		24 (64.9%)	13 (35.1%)		25 (67.6%)	12 (32.4%)	
Poor	160	75 (46.9%)	85 (53.1%)		74 (46.3%)	86 (53.8%)		73 (45.6%)	87 (54.4%)	
ER				0.001*			0.009*			0.009*
Negative	36	27 (75.0%)	9 (25.0%)		25 (69.4%)	11 (30.6%)		25 (69.4%)	11 (30.6%)	
Positive	161	72 (44.7%)	89 (55.3%)		73 (45.3%)	88 (54.7%)		73 (45.3%)	88 (54.7%)	
PR				0.692			0.617			0.424
Negative	69	36 (52.2%)	33 (47.8%)		36 (52.2%)	33 (47.8%)		37 (53.6%)	32 (46.4%)	
Positive	128	63 (49.2%)	65 (50.8%)		62 (48.4%)	66 (51.6%)		61 (47.7%)	67 (52.3%)	
Ki-67 index(%)				0.352			0.176			<0.001*
<30	93	50 (53.8%)	43 (46.2%)		51 (54.8%)	42 (45.2%)		59 (63.4%)	34 (36.6%)	
≥30	104	49 (47.1%)	55 (52.9%)		47 (45.2%)	57 (54.8%)		39 (37.5%)	65 (62.5%)	
Ascites				0.599			0.661			0.447
No	53	25 (47.2%)	28 (52.8%)		25 (47.2%)	28 (52.8%)		24 (45.3%)	29 (54.7%)	
Yes	144	74 (51.4%)	70 (48.6%)		73 (50.7%)	71 (49.3%)		74 (51.4%)	70 (48.6%)	
Lymph node metastasis				0.112			0.211			0.082
Negative	78	42 (53.8%)	36 (46.2%)		41 (52.6%)	37 (47.4%)		43 (55.1%)	35 (44.9%)	
Positive	62	25 (40.3%)	37 (59.7%)		26 (41.9%)	36 (58.1%)		25 (40.3%)	37 (59.7%)	
Unknown	57									

*p < 0.05, statistically significant.

a Using Chi-square test, p < 0.05 was considered statistically significant.

ER, estrogen receptor; PR, Progesterone receptor.

### Association Between Inflammatory Markers and Tumor-Infiltrating T Cells

**Figure 3:**
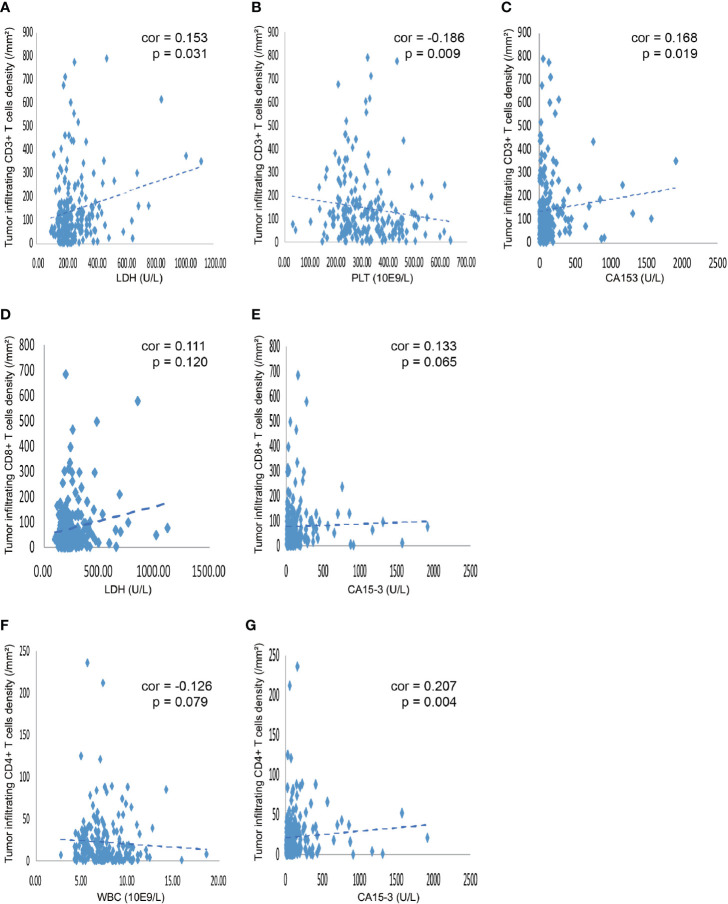
Correlation between various blood indicators and the density of TILs. **(A)** The density of CD3+ TILs showed a positive correlation with the level of serum LDH, with a coefficient of 0.153 (p = 0.031). **(B)** The density of CD3+ TILs was negatively related to the PLT, with a coefficient of −0.186 (P = 0.009). **(C)** The density of CD3+ TILs showed a positive correlation the level of serum CA153, with a coefficient of 0.168 (P = 0.019). **(D)** The density of CD8+ TILs showed a tendency that positively correlated with the level of serum LDH, with a coefficient of 0.111 (P = 0.120). **(E)** The density of CD8+ TILs showed a tendency that positively related to the serum CA153, with a coefficient of 0.133 (P = 0.065). **(F)** The density of CD4+ TILs showed a tendency that negatively correlated with the number of WBC in the blood, with a coefficient of -0.126 (P = 0.079). **(G)** The density of CD4+ TILs was positively related to the CA153, with a coefficient of 0.207 (P = 0.004). TILs, tumor-infiltrating lymphocytes; WBC, white blood cells; LDH, lactate dehydrogenase; PLT, platelet; CA153, carbohydrate antigen 153.

As shown in **Table 3**, the levels of LDH, PLT, and WBC demonstrated a significant association with the expression levels of TILs. Furthermore, we investigated the linear relationship between the three inflammatory markers and TILs, and found that higher level of serum LDH was associated with a higher density of CD3+ TILs in the tumor microenvironment, with a coefficient of 0.153 (p = 0.031) (**Figure 3A**). **Figure 3B** presents that the more PLT was significantly associated with lower levels of CD3+ TILs in the tumor, with a coefficient of −0.186 (P = 0.009). However, no significant linear correlation between LDH level and density of CD8+ TILs was observed, with a coefficient of 0.111 (p = 0.120) (**Figure 3D**) and the same for the correlation between WBC and CD4+ TILs density, with a coefficient of −0.126 (P = 0.079) (**Figure 3F**). We also found that the density of TILs had no significant correlations with neutrophils, lymphocytes, monocytes, NLR, PLR, LMR, ALB, and CRP in the tumor microenvironment (**Table 3**).

**Table 3:** Association between inflammatory markers and T cells in the microenvironment.

Variable	CD3+ T cells			CD8+ T cells			CD4+ T cells		
	Low	High	p-value^a^	Low	High	p-value^a^	Low	High	p-value^a^
WBC	7.20 (4.30-15.90)	7.20 (2.70-18.60)	0.396	7.05 (4.40-15.90)	7.30 (2.70-18.60)	0.898	7.50 (2.70-18.60)	6.80 (4.20-14.20)	0.026*
Neutrophils	4.90 (1.30-13.98)	4.75 (2.00-15.70)	0.247	4.89 (1.30-13.98)	4.90 (2.00-15.70)	0.762	5.17 (2.00-15.70)	4.70 (1.30-11.02)	0.075
Lymphocytes	1.50 (0.60-3.39)	1.70 (0.20-3.00)	0.220	1.50 (0.60-3.39)	1.70 (0.20-3.00)	0.093	1.50 (0.20-3.39)	1.60 (0.60-2.80)	0.932
Monocytes	0.41 (0.10-1.20)	0.42 (0.20-1.10)	0.753	0.41 (0.10-1.20)	0.44 (0.20-1.10)	0.865	0.50 (0.20-1.20)	0.40 (0.10-1.07)	0.284
Platelets	317.00 (30.50-637.00)	285.00 (132.00-614.00)	0.021*	310.50 (30.50-637.00)	294.00 (132.00-614.00)	0.286	309.00 (30.50-637.00)	299.00 (41.00-614.00)	0.490
NLR	3.50 (0.57-18.83)	2.91 (0.97-12.08)	0.191	3.48 (0.57-18.83)	2.92 (0.93-12.08)	0.264	3.46 (0.89-18.83)	2.94 (0.57-15.83)	0.326
PLR	208.46 (20.33-950.75)	177.71 (64.33-1155.00)	0.062	204.02 (20.33-950.75)	177.26 (64.33-1155.00)	0.145	202.36 (20.33-1155.00)	185.64 (27.33-641.25)	0.893
LMR	3.48 (0.92-12.96)	3.71 (0.67-10.00)	0.357	3.41 (0.92-10.27)	3.67 (0.67-12.96)	0.253	3.49 (0.67-12.96)	3.67 (0.98-10.00)	0.354
LDH	214.00 (97.90-652.70)	237.45 (118.40-1117.70)	0.032*	213.40 (97.90-652.70)	236.50 (118.40-1117.70)	0.021*	216.50 (114.20-761.90)	234.00 (97.90-1117.70)	0.208
ALB	41.90 (20.70-52.20)	41.35 (25.40-50.70)	0.754	42.05 (20.70-52.20)	41.50 (25.40-48.30)	0.418	42.45 (27.30-52.20)	40.90 (20.70-50.70)	0.206
CRP	12.24 (0.35-218.29)	7.56 (0.22-151.28)	0.090	7.44 (0.22-218.29)	11.55 (0.27-151.28)	0.968	10.01 (0.35-218.29)	8.14 (0.22-179.28)	0.418

*p < 0.05, statistically significant.

a Using Nonparametric test, p < 0.05 was considered statistically significant.

WBC, white blood cells; NLR, neutrophil-to-lymphocyte ratio; PLR, platelet-to-lymphocyte ratio; LMR, lymphocyte-to-monocyte ratio; LDH, lactate dehydrogenase; ALB, albumin; CRP, C-reactive protein.

### Association Between Tumor Markers and Tumor-Infiltrating T Cells

Only CA153 was significantly associated with the density level of TILs, as displayed in **Table 4**. Linear correlation analysis showed that higher level of serum CA153 was associated with higher density of CD3+ or CD4+ TILs in the tumor microenvironment with a coefficient of 0.168 (p = 0.019) and 0.207 (p = 0.004) (**Figures 3C, G**), respectively. However, a similar trend for CD8+ TILs was not observed, which had a coefficient of 0.133 (p = 0.065). The remaining tumor markers, including CA125, CEA, and CA199, had no significant association with the density of TILs.

**Table 4:** Association between tumor markers and tumor infiltrating T cells.

Variable	CD3+ T cells			CD8+ T cells			CD4+ T cells		
	Low	High	p-value^a^	Low	High	p-value^a^	Low	High	p-value^a^
CA125	738.1 (10.12-17009.00)	984.95 (3.48-18277.00)	0.576	747.05 (10.12-17009.00)	974.10 (3.48-18277.00)	0.335	770.40 (9.60-17009.00)	958.90 (3.48-18277.00)	0.277
CA153	45.16 (4.69-912.90)	82.11 (7.33-1918.00)	0.011*	46.49 (4.69-1574.00)	75.32 (7.33-1918.00)	0.065	45.53 (6.54-1313.00)	85.90 (4.69-1918.00)	0.016*
CA199	15.24 (0.60-4800.00)	11.26 (0.60-3537.00)	0.421	13.26 (0.60-4800.00)	12.53 (0.60-3537.00)	0.879	15.63 (0.60-4800.00)	11.54 (0.60-3537.00)	0.161
CEA	1.46 (0.20-1832.00)	1.30 (0.20-153.80)	0.187	1.40 (0.20-1832.00)	1.35 (0.20-153.80)	0.642	1.41 (0.20-1832.00)	1.35 (0.20-153.80)	0.250

*p < 0.05, statistically significant.

a Using Nonparametric test, p < 0.05 was considered statistically significant.

CA125, carbohydrate antigen 125; CA153, carbohydrate antigen 153; CA199, carbohydrate antigen 199; CEA, carcinoembryonic antigen.

### Independent Predictors of Density Levels of TILs

Our previous results showed that age, menopausal status, pathological differentiation, ER, Ki-67 index, CA153, WBC, PLT, and LDH had significant association with the expression levels of TILs. To further distinguish independent predictors of expression levels of TILs, we assessed the aforementioned clinical characteristics and blood indicators using the binary logistic regression. Predictors for CD3+, CD8+, CD4+ TILs were initially distinguished by univariate logistic regression analysis (**Table 5**). Six variables, including menopausal status, pathological differentiation, ER, CA153, PLT, and LDH, were potentially associated with the expression levels of CD3+ TILs (p < 0.1). Age, menopausal status, pathological differentiation, ER, and LDH were potentially associated with expression levels of CD8+ TILs (p < 0.1). Additionally, there were six potential factors related to the expression levels of CD4+ TILs, namely, menopausal status, pathological differentiation, ER, Ki-67 index, CA153, and WBC. Then, all of the above factors were included in multivariate regression analyses to identify the respective independent predictors of CD3+, CD8+, and CD4+ TILs (**Table 6**). Three factors were actually correlated with the expression level of CD3+ TILs: ER [positive: odds ratio (OR) 4.455, 95% CI 1.761–11.274, p = 0.002], PLT (234.7≤ x <305.0: 0.661, 0.277–1.575, p = 0.350; 305.0≤ x <379.8: 0.800, 0.332–1.928, p = 0.619; ≥379.8: 0.151, 0.056-0.405, p < 0.001) and LDH (182.1≤ x <224.5: 1.715, 0.709–4.152, p = 0.232; 224.5≤ x <306.6: 1.713, 0.708–4.145, p = 0.233; ≥306.6: 4.885, 1.825–13.076, p = 0.002). There were also three factors correlated with the expression level of CD8+ TILs: Menopausal status (positive: 1.926, 1.047–3.544, p = 0.035), ER (positive: 2.771, 1.199–6.402, p = 0.017), and LDH (182.1≤ x <224.5: 1.576, 0.669–3.710, p = 0.298; 224.5≤ x <306.6: 1.659, 0.704–3.907, p = 0.247; ≥306.6: 3.654, 1.450–9.209, p = 0.006). Similarly, the expression levels of CD4+ TILs were related to four factors: ER (positive: 2.400, 1.031–5.584, p = 0.042), Ki-67 index (≥30%: 3.034, 1.604–5.741, p = 0.001), CA153 (22.3≤ x <62.1: 1.151, 0.470–2.817, p = 0.759; 62.1≤ x <148.1: 1.557, 0.629–3.856, p = 0.339; ≥148.1: 3.479, 1.385–8.743, p = 0.008), and WBC (5.95≤ x <7.2: 0.347, 0.141–0.855, p = 0.022; 7.2≤ x <8.8: 0.281, 0.115–0.688, p = 0.005; ≥8.8: 0.219; 0.086–0.559, p = 0.002).

**Table 5:** Correlative factors for tumor infiltrating T cells identified by univariate logistic regression analysis.

Variable	CD3+ T cells			CD8+ T cells			CD4+ T cells		
	OR	95%CI	p-value	OR	95%CI	p-value	OR	95%CI	p-value
Age(year)									
<52	1			1			1		
≥52	0.679	0.387–1.190	0.176	1.809	1.029–3.183	0.040*	1.200	0.686–2.100	0.523
Menopausal status									
Negative	1			1			1		
Positive	0.619	0.351–1.092	0.098	2.161	1.217–3.837	0.009*	1.676	0.950–2.957	0.075
Pathological differentiation									
Moderate	1			1			1		
Poor	2.092	0.995–4.398	0.051	2.146	1.021–4.511	0.044*	2.483	1.167–5.284	0.018*
ER									
Negative	1			1			1		
Positive	3.708	1.640–8.385	0.002*	2.740	1.263–5.941	0.011*	2.740	1.263–5.941	0.011*
Ki-67 index(%)									
<30	1			1			1		
≥30	1.305	0.745–2.287	0.352	1.473	0.839–2.583	0.177	2.892	1.620–5.162	<0.001*
CA153 (U/ml)									
<22.3	1			1			1		
22.3≤ x <62.1	1.105	0.494–2.475	0.808	0.788	0.353–1.756	0.560	1.300	0.583–2.901	0.521
62.1≤ x <148.1	1.804	0.802–4.057	0.154	1.519	0.680–3.398	0.308	1.659	0.738–3.728	0.220
≥148.1	2.410	1.066–5.447	0.034*	1.576	0.707–3.512	0.266	2.629	1.158–5.967	0.021*
WBC (10E9/L)									
<5.95	1			1			1		
5.95≤ x <7.2	0.556	0.247–1.248	0.154	0.506	0.224–1.142	0.101	0.427	0.187–0.976	0.044*
7.2≤ x <8.8	0.780	0.355–1.716	0.537	0.917	0.417–2.014	0.828	0.368	0.163–0.831	0.016*
≥8.8	0.692	0.313–1.529	0.363	0.957	0.434–2.110	0.912	0.381	0.168–0.863	0.021*
Platelets (10E9/L)									
<234.7	1			1			1		
234.7≤ x <305.0	0.662	0.298–1.472	0.312	1.085	0.491–2.398	0.840	1.278	0.578–2.828	0.544
305.0≤ x <379.8	1.125	0.502–2.521	0.774	1.440	0.650–3.192	0.369	1.128	0.513–2.482	0.764
≥379.8	0.276	0.119–0.640	0.003*	0.557	0.249–1.249	0.156	0.849	0.384–1.878	0.686
LDH (U/L)									
<182.1	1			1			1		
182.1≤ x <224.5	1.516	0.679–3.382	0.310	1.516	0.679–3.382	0.310	0.921	0.416–2.039	0.839
224.5≤ x <306.6	1.457	0.656–3.240	0.355	1.579	0.711–3.509	0.262	1.043	0.474–2.297	0.916
≥306.6	2.719	1.201–6.156	0.016*	2.719	1.201–6.156	0.016*	1.785	0.800–3.985	0.157

*p < 0.05, statistically significant.

ER, estrogen receptor; CA153, carbohydrate antigen 153; WBC, white blood cells; LDH, lactate dehydrogenase; OR, odds ratio; 95% CI, 95% confidence interval.

**Table 6:** Correlative factors for tumor infiltrating T cells identified by multivariate logistic regression analysis.

Variable	CD3+ T cells			CD8+ T cells			CD4+ T cells		
	OR	95%CI	p-value	OR	95%CI	p-value	OR	95%CI	p-value
Age(year)									
<52	—			—			—		
≥52	—			—			—		
Menopausal status									
Negative	—			1			—		
Positive	—			1.926	1.047–3.544	0.035*	—		
Pathological differentiation									
Moderate	—			—			—		
Poor	—			—			—		
ER									
Negative	1			1			1		
Positive	4.455	1.761–11.274	0.002*	2.771	1.199–6.402	0.017*	2.400	1.031–5.584	0.042*
Ki-67 index(%)									
<30	—			—			1		
≥30	—			—			3.034	1.604–5.741	0.001*
CA153 (U/ml)									
<22.3	—			—			1		
22.3≤ x <62.1	—			—			1.151	0.470–2.817	0.759
62.1≤ x <148.1	—			—			1.557	0.629–3.856	0.339
≥148.1	—			—			3.479	1.385–8.743	0.008*
WBC (10E9/L)									
<5.95	—			—			1		
5.95≤ x <7.2	—			—			0.347	0.141–0.855	0.022*
7.2≤ x <8.8	—			—			0.281	0.115–0.688	0.005*
≥8.8	—			—			0.219	0.086–0.559	0.002*
Platelets (10E9/L)									
<234.7	1			—			—		
234.7≤ x <305.0	0.661	0.277–1.575	0.350	—			—		
305.0≤ x <379.8	0.800	0.332–1.928	0.619	—			—		
≥379.8	0.151	0.056–0.405	<0.001*	—			—		
LDH (U/L)									
<182.1	1			1			—		
182.1≤ x <224.5	1.715	0.709–4.152	0.232	1.576	0.669–3.710	0.298	—		
224.5≤ x <306.6	1.713	0.708–4.145	0.233	1.659	0.704–3.907	0.247	—		
≥306.6	4.885	1.825–13.076	0.002*	3.654	1.450–9.209	0.006*	—		

*p < 0.05, statistically significant.

ER, estrogen receptor; CA153, carbohydrate antigen 153; WBC, white blood cells; LDH, lactate dehydrogenase; OR, odds ratio; 95% CI, 95% confidence interval.

### Development and Validation of Nomograms for Density Levels of TILs

**Figure 4:**
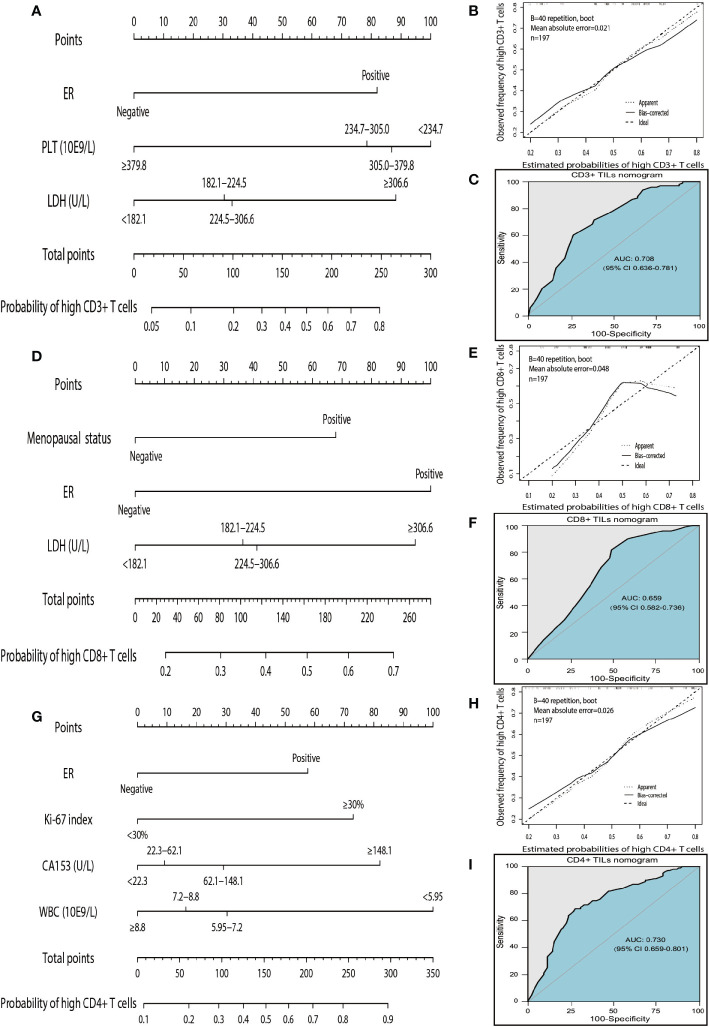
Nomograms, calibration curves, and ROC curves analysis for predicting density of TILs in patients with high-grade serous ovarian cancer. **(A)** The CD3+ TILs prediction nomogram. **(B)** Calibration curves for predicting density of CD3+ TILs. **(C)** ROC curves of CD3+ TILs prediction nomogram in the internal testing set. **(D)** The CD8+ TILs prediction nomogram. **(E)** Calibration curves for predicting density of CD8+ TILs. **(F)** ROC curves of CD8+ TILs prediction nomogram in the internal testing set. **(G)** The CD4+ TILs prediction nomogram. **(H)** Calibration curves for predicting density of CD4+ TILs. **(I)** ROC curves of CD4+ TILs prediction nomogram in the internal testing set. All the points assigned on the top point scale for each factor are summed together to generate a total point score. The total point score is projected on the bottom scales to determine the probability of high density for tumor-infiltrating T cells in an individual. The nomogram-predicted frequency of high T cell density is plotted on the x-axis, and the actual observed frequency of high T cell density is plotted on the y-axis. The AUC was calculated, and its 95% CI was estimated by bootstrapping. TILs, tumor-infiltrating lymphocytes; ROC, receiver operating characteristic; 95% CI, 95% confidence interval.

According to the independent predictors identified in the multivariate logistic regression analysis, three nomograms were respectively developed to predict the possible density levels of CD3+ (**Figure 4A**), CD8+ (**Figure 4D**), and CD4+ (**Figure 4G**) TILs in patients with HGSOC. The CD3+ TILs nomogram showed that PLT had the largest contribution, followed by LDH and ER. ER made the largest contribution in the CD8+ TILs nomogram, followed by LDH and menopausal status. For the CD4+ TILs nomogram, WBC made the largest contribution, followed by CA153, Ki-67 index, and ER. Harrell' concordance indicators of CD3+ (C-index = 0.748), CD8+ (C-index = 0.683), and CD4+ (C-index = 0.759) TILs nomograms were assessed and the calibration curves showed their good agreement between predictions and observations (**Figures 4B, E, H**). Then, we applied ROC analysis to evaluate the discrimination power for TILs nomograms. In the ROC curves of CD3+ TILs nomogram, the AUC value was 0.708 (95%CI 0.636–0.781) (**Figure 4C**), for the CD8+ TILs nomogram, it was 0.659 (95%CI 0.582–0.736) (**Figure 4F**) and was 0.730 (95%CI 0.659–0.801) (**Figure 4I**) for the CD4+ TILs nomogram. The results show that the CD4+ TILs nomogram had the best calibration and discrimination, followed by CD3+ and CD8+ TILs nomograms.

The instruction for using nomograms are as follows. The score of the parameter is displayed at the top of the scale, and sum up the scores of each parameter. Finding the corresponding point at the axis of total points and drawing a line perpendicularly to the axis of high TILs infiltration probability, the intersection point is the personal probability of high TILs infiltration. For example, for high CD3 T cells, values for negative ER, PLT<234.7 10E9/L, and LDH <182.1 U/L will obtain a total score of approximately 100 which means the probability for high CD3+ T cells infiltration is 0.2. It implies that the patient has a high probability of lacking of CD3+ T cells infiltration.

## Discussion

We present a retrospective study with TMA analysis, to explore the correlations of TILs (CD3+, CD8+, or CD4+) with clinical characteristics and blood indicators in HGSOC; based on which novel nomograms to help monitor the density levels of TILs in the tumor environment were developed. Our study showed the older patients in menopause had more CD8+ TILs and the clinical characteristics (including pathological differentiation, ER, and Ki-67) were significantly associated with TILs in HGSOC patients. Additionally, inflammation markers and tumor markers including WBC, PLT, LDH, and CA153 demonstrated significant linear correlations with TILs. Multivariate analysis indicated that most of the above markers were independent predictors for TILs and the nomograms displayed good efficacy to assess the density levels of CD3+, CD8+, and CD4+ TILs in the tumor environment. These results imply that the nomograms using minimally-invasive peripheral blood markers could be a promising approach to facilitate the monitoring of the immune status for HGSOC patients.

We observed more CD8+ TILs in older or menopausal patients than those in younger or un-menopausal patients but no similar differences for CD3+ and CD4+ TILs were found. We hypothesized that this might be because the state of the patient's hormone levels may play a more important role in immune function than age. There was a bi-directional interaction effect for hormones and immune system (32) and fertile women were found to be more vulnerable to auto-immune diseases than men till post-menopause (33). Kumru and his colleagues reported that surgical menopause contributed to an increase in CD8+ cells and reduced the ratio of CD4+ to CD8+ cells, and such trends could be reversed by estrogen replacement therapy (34). Therefore, estrogen deficiency may be the cause for the change of lymphocyte subsets in the tumor microenvironment. In our study, we stratified the patients based on the median age of 52 years. The median menopausal age of Chinese women reported was 50 years (35). Thus, we observed an increased CD8+ TILs in both older and post-menopausal people. Additionally, the interaction process of tumor cells and the immune system is critical for tumor progression. The infiltration of various TILs in the tumor microenvironment reflects the immune response to tumor aggressive biological features. The present study revealed that the increased number of various TILs was significantly associated with pathological differentiation and ER status, and the increased number of CD4+ TILs was significantly associated with high expression of the Ki-67 index (p < 0.001) (**Table 2**). The relationships between TILs and biological features of tumors shown here are in line with the results from other literatures in ovarian and breast cancers (36, 37). It would be meaningful to further explore the correlations and potential mechanisms between TILs and tumor pathological characteristics in a large number of patients with HGSOC.

Several studies have demonstrated that high levels of pro-inflammatory cytokines and inflammation among HGSOC patients were associated with the initiation and/or progression of most ovarian cancers (38, 39). We observed that the concentration of various TILs was closely correlated to the inflammatory markers. High densities of CD3+ and CD8+ TILs in the tumor microenvironment were associated with higher levels of serum LDH. Besides, the levels of PLT and WBC were negatively related to the densities of CD3+ and CD4+ TILs, respectively. The high level of serum LDH could suppress immune function in the tumor microenvironment and was a poor prognostic biomarker for many solid neoplasms (40–42). Previous study reported that the high level of lactate and acidification promoted immune inactivation and immune escape, even though tumor tissue with heavily infiltrated by functionally inactive T cells in metastatic melanomas (43). Cancer-associated thrombocytosis is a poor prognostic factor for various cancer types and platelets also have adverse effects on adaptive immunity (44). Another study showed that the TGF-β released by PLT could suppress anti-tumor T cell immunity and promoted tumor immune escape (45). Especially, increased counts of WBC also showed a heightened inflammatory state (46). A previous study showed that CD4+ T regulatory cells could inhibit neutrophil function and promote their apoptosis and death (47), and our results also pointed out a negative relation trend of neutrophil levels and CD4 + TILs (**Table 3**). We speculated that the WBC count was the sum of the absolute numbers of various inflammatory cells, so the WBC count was more likely to be an overall reflection of the association between CD4+ TILs and various inflammatory cells.

We observed that the level of CA153 in the blood was positively correlated with the densities of CD3+ and CD4+ TILs in the tumor microenvironment, and the same tendency for CD8+ TILs (**Table 4**) was observed. However, CA153 was an independent predictor only for CD4+ TILs in the multivariate logistic analysis and other tumor markers, such as CA125, CEA, CA199, had no significant association with TILs. Mucin 1 (MUC1), also known as CA153, is one of the commonly used tumor markers in the diagnosis and recurrence monitoring of ovarian cancer (48). The expression of MUC1 can disrupt cell–cell and cell–matrix adhesions, and promote tumor adhesion and presumably metastasis (49, 50). A previous study also showed soluble MUC1 mediated immune suppression by blocking T-cell activation (51) and Budiu et al. reported that the expression of MUC1 in ovarian tumor cells could promote regional spread and increase the accumulation of CD4+Foxp3+ immune-suppressive regulatory T cells which is often accompanied with high levels of MUC1 in the blood (52). Further investigation on circulating MUC1 for the chemotaxis of T cells in the tumor microenvironment would be interesting and the intensive molecular mechanisms are worthy of more elaborate study.

For advanced patients with HGSOC, to the approaches of preferentially select neoadjuvant chemotherapy (NAC) or primary debulking surgery remains controversial (53, 54). Besides prognostic value, higher pre-treatment TILs levels were related with higher pathologic complete response (pCR) for NAC (55). However, tumors lacking TILs tended to lack TILs after NAC, and the assessment of TILs level in pretreatment could help identify immune-inert tumors that would be probably resistant to NAC or immunotherapy (56). Conversely, tumors that initially have high TILs infiltration tend to have more TILs infiltration after NACT (57). Tumors with abundant TILs infiltration may have higher levels of PD-L1 expression, and patients may respond better to PD1/PD-L1 inhibitors (58). A study reported that before chemotherapy, patients with suppressed immune function were at high Treg levels, and low levels of cytotoxic, helper T cells and NK cells. However, a single cycle of the combination of carboplatin and paclitaxel can reverse the immunosuppressive effect, reaching a peak two weeks after treatment, suggesting that 1–2 days after chemotherapy is the best time to start immunotherapy (59, 60). Therefore, physicians could determine whether the patient needs to undergo NACT first to adjust the immune function and the timing of immunotherapy based on the status of tumor TILs infiltration. At present, TILs in tumor tissues are mainly evaluated by IHC staining of tissue sections (61). However, in daily clinical practice, this approach is invasive, costly and technically complex and alternatives are urgently warranted for timely treatment (62). Therefore, we focused on clinical research according to clinically easily accessible indicators. Some researchers have estimated the survival probability and therapeutic response using nomograms with multiple clinicopathologic factors. A study of 840 patients with epithelial ovarian cancer assessed available clinicopathological characteristics to develop nomograms for 5-year survival probability and showed accurate calibration and the C-index was 0.71 (95% CI 0.69–0.74) (63). In addition, Seung-Hyuk Shim et al. formulated and validated a positron-emission tomography/computed tomography-based nomogram incorporating the radiomics features and surgical aggressiveness index to facilitate the preoperative individualized prediction of incomplete cytoreduction in advanced ovarian cancer patients (64). Hye Won Hwang and colleagues developed a breast cancer therapeutic response nomogram to predict pCR based on pre-NAC TILs levels (65). Thus, nomograms are common prediction tools in oncology and the measuring scales with multiple factors are convenient for simple calculations, we decided to formulate nomograms for TILs prediction in HGSOC. Three nomograms were respectively developed and validated for predicting CD3+, CD8+, CD4+ TILs in patients with HGSOC. The nomogram for CD3+ TILs comprised of ER, PLT and LDH and the nomogram for CD8+ TILs incorporates menopausal status, ER and LDH, while the nomogram for CD4+ TILs contains four factors: ER, Ki-67, CA153, and WBC. The factors included in the nomograms were significantly associated with TILs in tumor microenvironment and all of the nomograms demonstrated favorable validation and discrimination in the internal verification. After investigated the correlations of densities of TILs with clinicopathologic characteristics and blood indicators in the tumor microenvironment, we proposed that menopausal status, ER, Ki-67 index, CA153, WBC, PLT, and LDH may be utilized to assess the immune status in HGSOC patients and these data could be easily acquired in the process of diagnosis through routine blood test and pathological biopsy. Importantly, the developed nomograms could greatly facilitate to simplify the calculation process in the assessment of the patient's immune status.

Nevertheless, the present study also had a few limitations. First, the nomograms lacked external validation. Because the sample size was limited for a single-center clinical retrospective research, multi-center large sample size verification could provide a higher level of evidence for clinical application. Second, this study did not incorporate genetic markers. Because genetic testing is not a routine examination, clinical factors and blood indicators were more frequently collected. However, combining genetic markers may improve the prediction of TILs nomograms in patients with HGSOC. Third, we did not analyze other types of TILs, such as B cells, natural killer cells, and other specific types of T cells, although these cells also played important roles in the tumor microenvironment. Fourth, the study did not pay attention to the location of TILs, which had particular significance in ovarian carcinoma. Due to the TMA method, the location and area of tumor tissue were restricted. Fifth, the conclusions of this study may only apply to high-grade serous ovarian cancer and the types of TILs are limited to CD3+, CD4+, and CD8+ T cells.

## Conclusions

Our results demonstrated that menopausal status, ER, Ki-67 index, CA153 level, WBC count, PLT, and LDH were associated with the densities of CD3+, CD8+ or CD4+ TILs in the tumor microenvironment. Based on the above factors identified, we developed the first applied nomograms that could conveniently assess individualized immune status of TILs for patients with HGSOC. Moreover, the nomograms demonstrated high accuracy and reliability in the internal validation and they could help clinicians to monitor patients' immune status and make clinical treatment strategies for patients with HGSOC.

## Data Availability Statement

The original contributions presented in the study are included in the article/supplementary material. Further inquiries can be directed to the corresponding authors.

## Ethics Statement

This study was approved by the Institutional Review Board and Ethics Committee at Sun Yat-sen University Cancer Center.

## Author Contributions

DD designed research. DD, LL, HH, SC, and BC analyzed the data and drafted the paper. SC, BC, and JC collected data. XL and FW contributed new reagents and analytic tools. LL, HH, SC, BC, and RL performed experiment research. RL and JL amended the paper. All authors contributed to the article and approved the submitted version.

## Funding

This work was supported by the National Natural Science Foundation of China (grant No.81772782 and No.81972443 to JL) and the High-level Hospital Construction Project (grant number DFJH201921, BC) and the Doctor Launch Fund of Guangdong Provincial People’s hospital (grant number 2018bq02, BC).

## Conflict of Interest

The authors declare that the research was conducted in the absence of any commercial or financial relationships that could be construed as a potential conflict of interest.

## Abbreviations

FIGO, International Federation of Gynecology and Obstetrics; HGSOC, High-grade serous ovarian cancer; TILs, Tumor-infiltrating lymphocytes; ROC, Receiver operating characteristic; AUC, Area under the curve; C-index, Harrell's concordance index; White blood cells, WBC; NLR, Neutrophil-to-lymphocyte ratio; PLR, Platelet-to-lymphocyte ratio; LMR, Lymphocyte-to-monocyte ratio; PLT, Platelet; LDH, Lactate dehydrogenase; CRP, C-reactive protein; ALB, Albumin; CA125, Carbohydrate antigen 125; CA153, Carbohydrate antigen 153; CEA, Carcinoembryonic antigen; CA 199, Carbohydrate antigen 199; ER, Estrogen receptor; PR, Progesterone receptor; IHC, Immunohistochemical; H&E, Hematoxylin and eosin; pCR, Pathologic complete response; NAC, Neoadjuvant chemotherapy.
